# Case of Fracture of the Upper Jaw-Bones

**Published:** 1859-09

**Authors:** C. S. Fenner

**Affiliations:** Memphis, Tennessee


ART. VII.—*A Case of Fracture of the Upper Jaw-Bones.* By C. S. FENNER, M.D., of Memphis, Tennessee.

FRANCIS PRATT, aged forty-five years, one of the injured survivors of the terrible explosion of the boilers of the steamer Pennsylvania, arrived in this city on the morning of the fourteenth of June, about twenty hours after the accident, and came under my charge, associated with Drs. Price and Hawkins. Mr. Pratt received a blow on the face from a fragment of the wreck, which divided the soft parts from the zygomatic arch to the centre of the upper lip, and separated the superior maxillary bones, together with the malar bone on the left side, from their bony attachments, except a part of the nasal processes, which were broken off, and remained in place. The upper jaws were separated at their junction, through the alveolar and palatine processes. From Dr. Burton, who was on the boat that brought the sufferers to Memphis, I learned that, when he first saw Mr. Pratt, the superior maxillary bones were turned outward, the grinding surfaces of the crowns of the teeth resting against the mucous membrane of the cheeks. Dr. Burton replaced the bones, but having no attachments except by mucous membrane, they dropped down against the lower jaw, and followed its motions. The inferior maxilla had an oblique fracture on the left side, between the lateral incisor and bicuspid teeth; and, although the articulations were uninjured, the bone remained considerably depressed, owing to the great swelling of the soft parts, and could not be brought to its natural position. The upper jaws were placed in apposition, and kept together by means of a wire twisted around the teeth. The lower jaw was treated in the same manner, a splint of binder's board adapted to the parts, and Barton's roller applied. A piece of cork was placed on each side, between the teeth, to keep the superior maxillary bones elevated to their natural position. At the end of three weeks they were so firmly united as to remain in place without the corks, and in six weeks firm bony union had taken place. The lower jaw did not so readily unite. An abscess formed near the fracture, and the parts became so painful that he could not bear the wire twisted sufficiently tight to hold the fractured ends of the bone together. The abscess was opened, and several spiculæ of bone were discharged, after which the wire was reapplied; but at the time of his leaving here, two months after the injury, bony union had not taken place.

This case is interesting, as it shows the great injury that may occur to the facial bones, and yet be followed by a speedy recovery.

